# Peri‐implant proximal femur fracture in a poliomyelitis survivor: A surgical and medical challenge

**DOI:** 10.1002/ccr3.7465

**Published:** 2023-06-05

**Authors:** Evangelos Sakellariou, Athanasios Galanis, Michail Vavourakis, Eftychios Papagrigorakis, Christos Vlachos, Dimitrios Zachariou, Elias Vasiliadis, Spiros Pneumaticos

**Affiliations:** ^1^ 3rd Department of Orthopaedic Surgery National & Kapodistrian University of Athens KAT General Hospital Athens Greece

**Keywords:** ORIF, peri‐implant femoral fracture, peri‐implant infection, poliomyelitis, *Staphylococcus warnerii*

## Abstract

**Key Clinical Message:**

The treatment of long bone fractures in post‐polio survivors is indubitably an exacting task. Out of this complicated case presented in this paper, it can be deduced that it is attainable to repair a peri‐implant subtrochanteric refracture or a complex non‐union of the proximal femur with plate and screws with grafting.

**Abstract:**

Post‐polio survivors are prone to low‐energy bone fractures. The management of such cases is exigent, as no literature data indicate the best surgical approach. This paper presents an intricate peri‐implant proximal femoral fracture in a *polio* survivor treated in our institution and accentuates the various challenges we encountered.

## 1 INTRODUCTION

*Poliomyelitis* is an infectious disease caused by *poliovirus*.^1^ Owing to globally established vaccination programs, the number of newly diagnosed polio cases has plunged, with the disease almost being eradicated in the United States after the use of vaccines.^1^, ^2^ However, *poliomyelitis* still occurs in underdeveloped countries because of the absence of an official vaccination protocol. Literature data support that an estimated population of 10–20 million have survived *poliomyelitis* infection and are still living with the disabling consequences of the disease.^1^ These patients cope daily with progressing paralysis of the lower extremities, muscle weakness without impaired sensation and limb length discrepancy. Although 70% of cases remain asymptomatic or present with mild symptoms that relent within 2–4 weeks, less than 1% of infections result in irreversible paralysis or even death.^1^ Poliovirus destroys the anterior horn cells in the spinal cord and the motor brainstem nuclei, spreading along certain nerve pathways.^3^, ^4^ The abovementioned fact results in these patients not presenting sensation abnormality, although they suffer from motor weakness. Furthermore, post‐polio syndrome, an associated condition, is a degenerative syndrome associated with aging, where more cells become inactive over time.^3^, ^4^ While no virus reactivation is detected, the syndrome occurs after middle age and affects up to 50% of previously infected patients.^2^, ^5^ Daily routine becomes more difficult as patients experience progressive exhaustion related to reduced muscle tone, but not at the point to cause muscle breakdown. Since the vaccine's introduction in the 1950s, the vast majority of patients who suffered from *poliomyelitis* in developed countries present satisfactory performance status in their sixth to seventh decade, but are susceptible to musculoskeletal complications, including osteoporotic fractures^2^, ^5^, ^6^ whose treatment is challenging and demanding. We report a rare case of a peri‐implant proximal femur fracture in a *poliomyelitis* survivor that has been treated surgically in our Institution and the challenges our team confronted during surgical and medical management.

## 2 CASE REPORT

An 82‐year‐old Caucasian female was admitted to the Emergency Department of our Institution complaining about acute right limb pain and inability to walk with full weight bearing due to a low‐impact fall.

**FIGURE 1:**
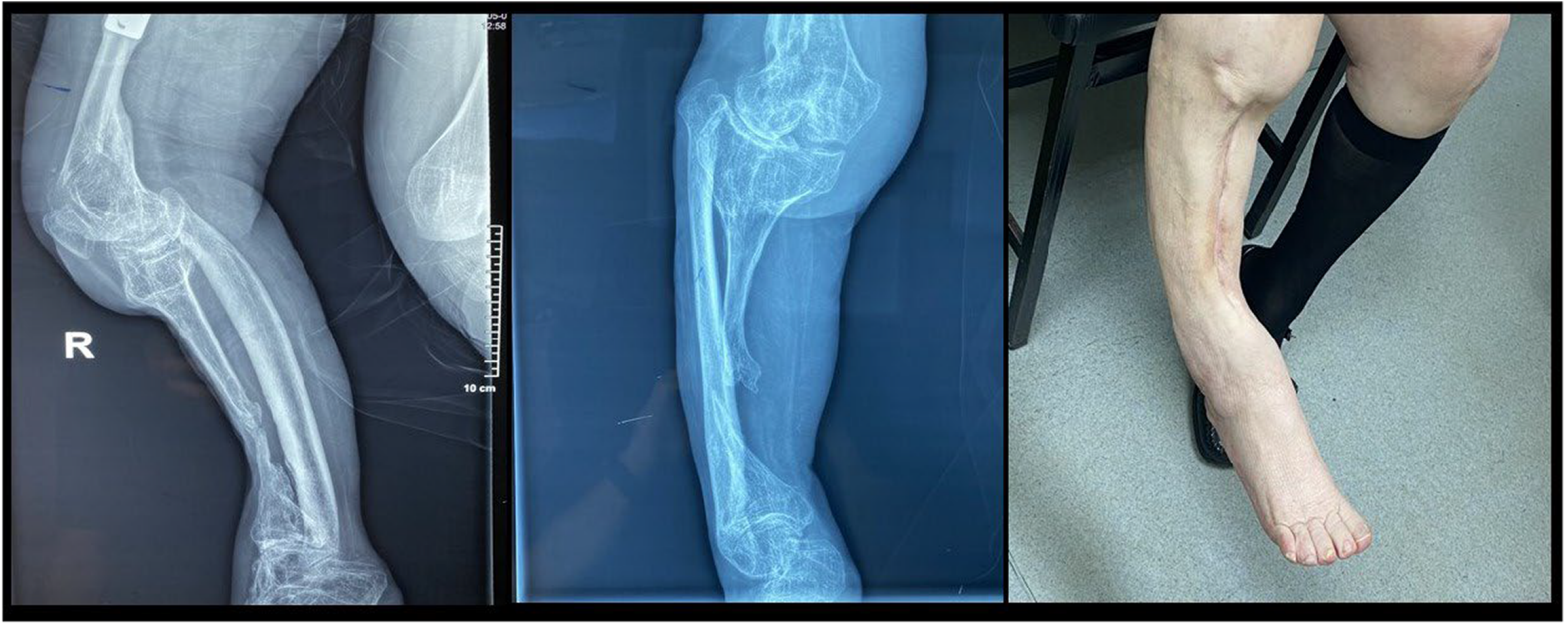
Radiographic imaging and clinical presentation.

Past medical history, apart from hypertension, dyslipidemia and osteoporosis also included a childhood poliomyelitis infection that had affected the ipsilateral lower limb. Post‐infection, the patient had a hypoplastic shin bone, deformed fibula and osteoporotic skeletal bones (Figure 1).

**FIGURE 2:**
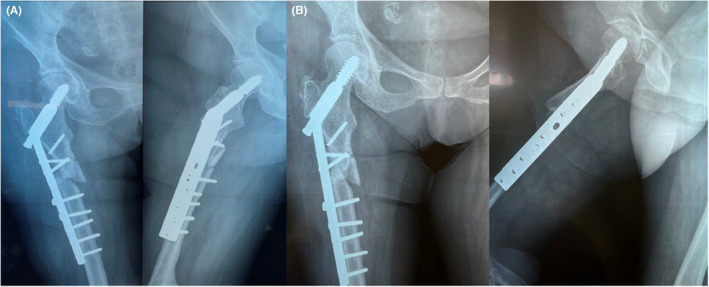
Dynamic hip screw (DHS) fixation of the initial subtrochanteric fracture (A) 1‐month post‐op and (B) 6‐month post‐op.

However, the patient was self‐sufficient in her daily routine, using only a walking stick and wearing permanent lower limb support. Three years ago, the patient sustained a traumatic intertrochanteric fracture after a low‐impact fall and underwent surgery in another hospital for open reduction and internal fixation (ORIF), where a dynamic hip screw (DHS) was implanted (Figure 2).

**FIGURE 3:**
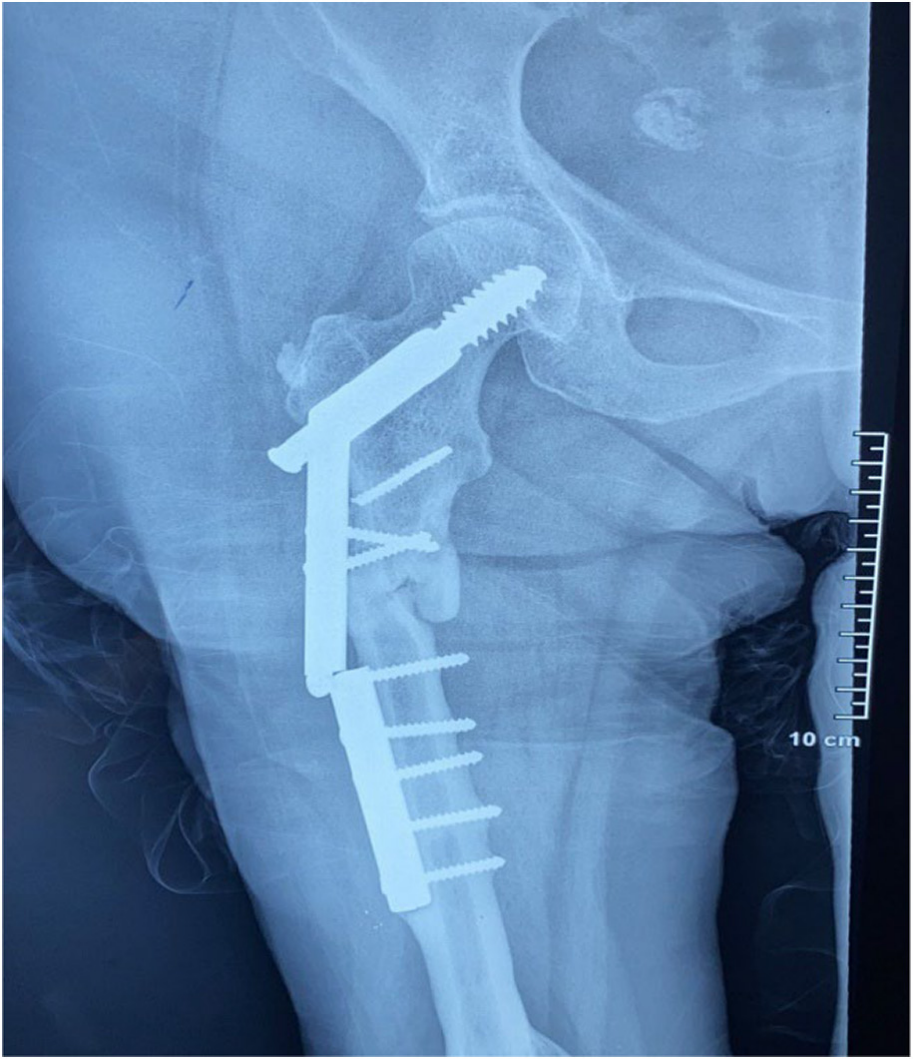
Proximal femoral peri‐implant fracture.

On clinical examination, the patient presented severe pain over the right hip and a significantly reduced range of motion. Her right limb was dystrophic and the ipsilateral quadriceps muscle showed signs of atrophy. Plain radiographic scans of the pelvis and hip were conducted to evaluate the anatomy of the hip, femur, tibia, and fibula and the integrity of the DHS. X‐rays revealed a peri‐implant fracture in the proximal femoral diaphysis involving the upper distal half of the internal fixation plate, which could presumably be attributed to a non‐union from the previous fixation (Figure 3).

Regarding laboratory workup, white blood cells count was 12.000/μl, erythrocyte sedimentation rate was 85 mm/h, and C‐reactive protein was 0.5 mg/dL (normal value < 0.31 mg/dL), which could be indicative of bone and soft tissue infection secondary to her previous hip surgery. This fact bolsters the supposition of a non‐union.

**FIGURE 4:**
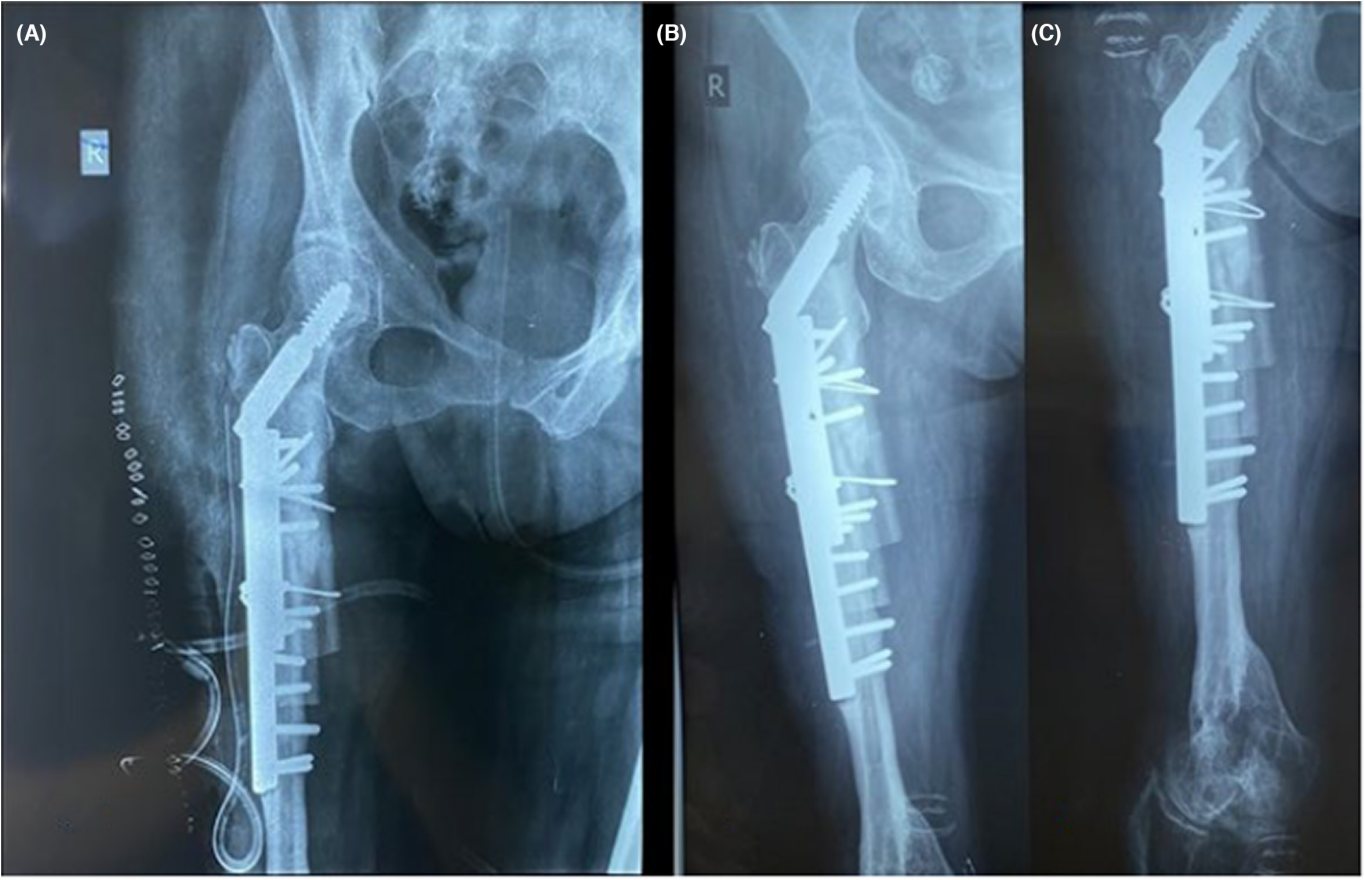
Postoperative radiographic imaging. (A) 1‐month post‐op, (B) 3‐month post‐op, and (C) 6‐month post‐op.

The patient underwent surgery under general anesthesia in the left lateral decubitus position using the same surgical approach as the previous operation. The distal half of the DHS plate and screws were removed and sent for analysis and sonication testing. The proximal half of the plate was found to be stable, and the intertrochanteric fracture was fully consolidated. Due to the poor quality of the patient's osteoporotic bone and to avoid major trauma, it was decided intraoperatively to keep the proximal half of the previous internal fixation in place. After removing the distal half of the plate and screws, the leading edges of the fracture were meticulously washed out. Soft tissue was removed from the fracture site and sent for microbiology examination. The fixation of the peri‐implant fracture was made by implanting a new diaphyseal plate and screws (4.5 mm plate with 10 holes), which were placed anteromedially, beginning proximally on the subtrochanteric area and finishing 7 cm below the fracture. The fixation was augmented with the use of a strut graft in the medial aspect of the femur, which was stabilized with the utilization of two cable wires (Figure 4). The surgical exposure was closed in a typical manner, and no intraoperative complications occurred.

Immediately postoperatively, intravenous teicoplanin was administered empirically. On the 3rd postoperative day, culture analysis revealed *Staphylococcus warnerii* infection, and based on sensitivity testing, antibiotic treatment was modified to cloxacillin.

## 3 CLINICAL OUTCOME

**FIGURE 5:**
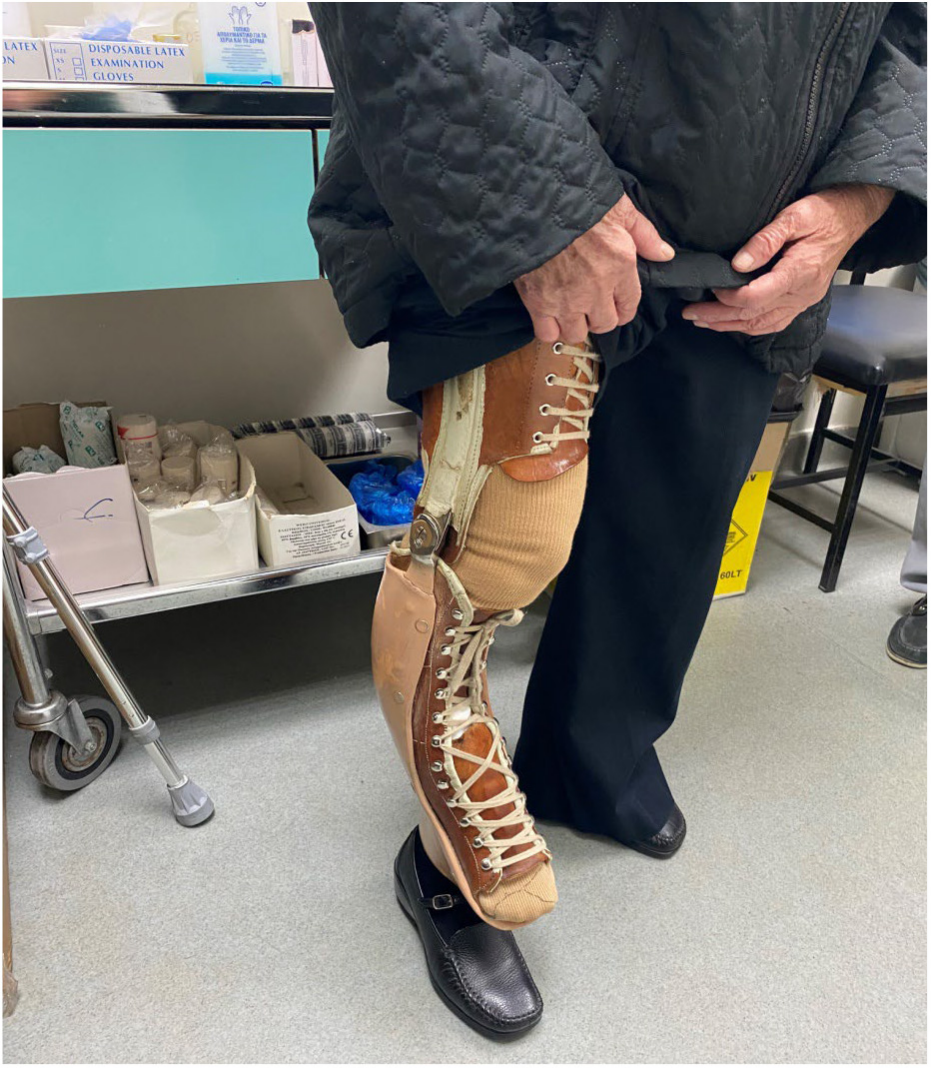
One year postoperatively.

On the 4th postoperative day, the patient began mobilization with the aid of a physiotherapist and an orthotic device, with no weight bearing of the affected limb. The patient was discharged after 15 days, with a per os antibiotic treatment regimen of rifampicin combined with sulfamethoxazole/trimethoprim, administered for two more weeks, following our department's infectious disease specialist's consultation. On the follow‐up examination, 2 months postoperatively, the patient was suggested to proceed with gradually increasing weight bearing of her right limb. 3‐month postoperative radiographs revealed good healing of the peri‐implant fracture (Figure 4). Neither evidence of implant loosening was detected nor signs of infection, and laboratory workup was normal. One year postoperatively, the patient had fully recovered, was fully weight bearing and independent with her activities of daily living (ADLs), only using a lower limb prosthesis and a walking stick (Figure 5).

## 4 DISCUSSION

Every poliomyelitis survivor, especially those with bone fractures, is an exacting case for an orthopedic surgeon. That derives from the fact that the bones of these patients are hypoplastic, deformed, and osteoporotic. Another problem correlated with polio infection is that because of impaired motor neurosis, muscle tone decreases, and the patient progressively paralyzes. Thus, the anatomy of bony components becomes tough in terms of surgical management.^5^, ^6^

In our case, four main challenges were encountered during the surgical management and healing. The first was the abnormal bone quality and anatomical structure that could not support a new entire ORIF. The second challenge was that our patient already had a previous ORIF with a DHS plate, which was intact proximally and fractured in the middle due to a new fall, whereas the likelihood of a non‐union was exceedingly high. In addition, concerns were raised regarding the type of internal fixation we were going to use to fix the new peri‐implant fracture. Last but not least was a peri‐implant infection caused by *S. warnerii*.

According to the literature, the incidence of bone fractures in poliomyelitis survivors accumulates with aging and ranges from 28% to 38%.^2^, ^5^, ^6^ The commonest fracture site occurs on the site of poliomyelitis involvement. Our patient sustained a proximal femur peri‐implant fracture on the limb previously affected by poliomyelitis infection. The high incidence of fracture in post‐polio patients may, in part, result from the high incidence of falls during ADLs associated with disability, which may be as high as 64% within 1 year and 79%–82% over 5 years.^7^ As in other findings, falls are the most frequent cause of the patients' fractures.

Literature supports that only 4% of poliomyelitis survivors have normal bone density, while 40% suffer from osteopenia, a risk factor for low‐energy bone fractures.^8^

Various implants and treatment methods are available to reduce and fix femoral fractures.^9^ Femoral canal diameter, femoral bowing, fracture location, and morphology and clinical deformities of the patients are key factors that determine the choice of fixation and implant.^9^, ^10^ Locking plates, pre‐contoured anatomical plates, and titanium elastic nailing systems offer an alternative in patients unsuitable for intramedullary nailing.^11^ In our case, we decided not to remove the previously placed DHS and stabilize the peri‐implant fracture with a new diaphyseal plate and screws, along with a strut graft supported by two cable wires. Our inference for implementing the plate and screws construct versus opting for intramedullary nailing was grounded on the fact that the large lag screw from the DHS was present and intact; therefore, it could be utilized for the new fixation. In addition, femoral bone quality was exceptionally poor while the medullary cavity was narrow, and finally the implementation of a nail could trigger a devastating impact regarding the high risk of concomitant infection. In post‐poliomyelitis patients, minimally invasive techniques and smaller incisions are required due to bone deformity, small femoral shafts, and medullary cavities.^12^, ^13^

During surgery, all pieces removed were sent for sonication analysis and cultures. Postoperatively, it was revealed that our patient suffered from a concomitant bone infection caused by *S. warnerii* and received targeted antibiotic therapy following sensitivity testing.

The clinical presentation of a bone infection, irrespectively of the pathogen, is most frequently associated with joint pain, accounting for 79%–100% of cases.^14^, ^15^ Other manifestations include local swelling, erythema, increased local temperature, and fever.^16^ The affected joint's range of motion is usually restricted due to pain and effusion.^17^ Pathophysiologically, most infections presented late after an operation involve the hematogenous route.^17^, ^18^ In suspicion of bone infections, literature data suggest that all patients should receive broad‐spectrum antibiotics for 2–4 weeks preoperatively and continue after surgery if cultures test positive, based on sensitivity testing.^19^, ^20^

## 5 CONCLUSION

This case presents an infrequent peri‐implant femoral fracture secondary to *poliomyelitis* and concomitant infection by *S. warnerii*. This case report aims to highlight the significance of stable fixation in every periprosthetic fracture, using the less possible fixation materials while eradicating bacterial bone infection, especially in high‐risk patients. The good result depends on the medical team's coordination, both intraoperatively and postoperatively, for the optimum outcome to be achieved.

## AUTHOR CONTRIBUTIONS

**Evangelos Sakellariou:** Data curation; software; writing – original draft. **Athanasios Galanis:** Conceptualization; data curation; formal analysis. **Michail Vavourakis:** Conceptualization; methodology; resources. **Eftychios Papagrigorakis:** Conceptualization; project administration; resources. **Christos Vlachos:** Formal analysis; investigation. **Dimitrios Zachariou:** Methodology; validation. **Elias Vasiliadis:** Visualization; writing – review and editing. **Spiros Pneumaticos:** Supervision.

## CONFLICT OF INTEREST STATEMENT

The authors declare no competing interests.

## CONSENT FOR PUBLICATION

The authors have obtained written consent for the publication of the data presented in this work.

## Data Availability

All raw data are available to access should they be requested.
